# Ocular adverse events associated with immune checkpoint inhibitors: a pharmacovigilance analysis of the FAERS database

**DOI:** 10.3389/fphar.2026.1808874

**Published:** 2026-07-27

**Authors:** Xin Liu, Ang Ji

**Affiliations:** Department of Ophthalmology, Beijing Haidian Hospital, Haidian Section of Peking University Third Hospital, Beijing, China

**Keywords:** Disproportionality analysis, FAERS, immune checkpoint inhibitors, ocular adverse events, pharmacovigilance, uveitis

## Abstract

**Background:**

To systematically characterize ocular adverse events (oAEs) associated with immune checkpoint inhibitors (ICIs) and to quantify their disproportionality reporting signals using real-world pharmacovigilance data.

**Methods:**

This retrospective pharmacovigilance study analyzed oAE reports associated with four ICI treatment regimens—anti-CTLA-4 monotherapy, anti-PD-1/L1 monotherapy, nivolumab plus ipilimumab combination therapy, and the novel nivolumab plus relatlimab combination—using the U.S. Food and Drug Administration Adverse Event Reporting System (FAERS) database from the first quarter of 2011 through the second quarter of 2025. Disproportionality analyses were conducted to detect potential safety signals employing three complementary metrics: reporting odds ratio (ROR), proportional reporting ratio (PRR), and information component (IC). Additional analyses included ophthalmologist-reviewed clinical grouping of uveitis subtypes, stratification by underlying tumor indication, and temporal trend assessment.

**Results:**

A total of 5,244 oAE reports associated with ICIs were identified. At the preferred term (PT) level, the strongest overall signals were predominantly inflammatory ocular conditions, including uveitis (ROR 6.04, 95% CI 5.45–6.69), scleritis (ROR 4.85, 95% CI 3.20–7.36), and optic neuritis (ROR 4.59, 95% CI 3.91–5.39). Combination therapy was associated with markedly stronger disproportionality signals; notably, the ROR for uveitis with nivolumab plus ipilimumab reached 15.44 (95% CI 13.24 to 18.00), compared with 4.37 for anti-PD-1/L1 monotherapy. The nivolumab plus relatlimab regimen showed preliminary signals for keratitis (ROR 13.82, 95% CI 3.44 to 55.61) and diplopia (ROR 9.59, 95% CI 3.07 to 29.91), though based on a very small sample (N = 18). Melanoma was disproportionately represented among uveitis-spectrum cases (53.6%), and temporal analysis revealed a declining uveitis proportion paralleling the expansion of ICI use beyond melanoma.

**Conclusion:**

ICIs are strongly associated with disproportionality signals for inflammatory ocular adverse events, with substantially higher signals observed for combination therapies compared with monotherapy. These hypothesis-generating findings highlight the need for prompt ophthalmologic assessment and individualized, multidisciplinary surveillance in patients receiving ICI treatment, particularly combined regimens.

## Introduction

Immune checkpoint inhibitors (ICIs) have revolutionized modern oncology, demonstrating durable clinical benefits across a wide spectrum of malignancies by augmenting the host anti-tumor immune response (Sun et al., 2024). By targeting inhibitory pathways such as cytotoxic T-lymphocyte-associated protein 4 (CTLA-4), programmed cell death protein 1 (PD-1), and programmed death-ligand 1 (PD-L1), ICIs unleash T cells to recognize and eliminate cancer cells. However, this generalized immune activation frequently leads to a unique spectrum of autoimmune toxicities known as immune-related adverse events (irAEs) (Iranzo et al., 2022; Li et al., 2025; Postow et al., 2018; Zhou et al., 2020; Lin et al., 2024). While irAEs most commonly affect the skin, gastrointestinal tract, and endocrine systems, toxicities in nearly every organ system have been reported (Zhou et al., 2020). Ocular adverse events (oAEs), though considered rare, represent a significant challenge due to their potential to cause severe, irreversible vision loss (Huillard et al., 2012). Manifestations such as uveitis, optic neuritis, and scleritis can profoundly impact a patient’s quality of life and may necessitate the cessation of life-saving cancer therapy (Lim et al., 2024).

The true incidence and reporting signal profile of oAEs are difficult to ascertain from randomized controlled trials (RCTs), which often have sample sizes and follow-up durations insufficient to detect rare adverse events. Consequently, post-marketing pharmacovigilance, utilizing large, real-world databases, is essential. The FDA Adverse Event Reporting System (FAERS), one of the world’s largest spontaneous reporting databases, provides a critical tool for detecting and analyzing potential drug safety signals that may be missed in clinical trials (Potter et al., 2025).

Previous foundational studies using FAERS have established an association between ICIs and oAEs. Seminal analyses by Fang et al. (2019) (10) and Bomze et al. (2020) (Bomze et al., 2022) provided the first large-scale quantifications, identifying uveitis as a primary signal using data up to 2018 and 2019, respectively. However, the landscape of ICI therapy has evolved rapidly. Data from the last 6 years, which includes the approval of new drug classes (e.g., the anti-LAG-3 agent Relatlimab) and the dramatically expanded use of combination therapies, have not been comprehensively analyzed (Ghasemi, 2025; Chen Z. et al., 2024). Furthermore, the expanding application of ICIs across non-melanoma indications may have altered the ocular toxicity profile, as the underlying tumor type itself is a relevant factor for ocular irAEs.

Therefore, this study aims to provide a systematic and updated analysis of ICI-associated oAEs by leveraging the most current FAERS data available (2011 Q1 to 2025 Q2). In addition to standard disproportionality signal detection stratified by treatment regimen, we performed ophthalmologist-reviewed clinical grouping of overlapping inflammatory entities, stratification by underlying cancer indication, and temporal trend analysis to evaluate how the pattern of oAE reporting has evolved alongside changes in ICI prescribing.

## Methods

### Data source and pre-processing

This retrospective pharmacovigilance study utilized data from the FAERS, a publicly available database designed to support the FDA’s post-marketing safety surveillance program. This study extracted data covering the period from the first quarter of 2011 (2011 Q1) to the second quarter of 2025 (2025 Q2) (U.S. Food and Drug Administration, 2024). The FAERS database comprises seven data files: demographic information (DEMO), drug information (DRUG), adverse reaction information (REAC), patient outcomes (OUTC), report sources (RPSR), therapy start and end dates (THER), and indications (INDI). These files were linked using unique case identification numbers (PRIMARYID) for data integration. Prior to analysis, a deduplication procedure was performed to ensure data quality. Following the recommendations of established pharmacovigilance protocols, reports with identical values for demographic (age, sex), administrative (event date, reporting country), and drug information were considered duplicates. In such cases, the most recent report was retained for analysis based on the FDA_DT (date FDA received the report). De-identified public data was used in this study, not requiring any form of ethics approval.

### Drug identification and cohort definition

Target drugs were identified by querying the FAERS DRUG file using both generic and brand names of FDA-approved ICIs. The drug search strategy encompassed all approved generic names (ipilimumab, nivolumab, pembrolizumab, atezolizumab, avelumab, durvalumab, tislelizumab, and toripalimab), their corresponding brand names (Yervoy, Opdivo, Keytruda, Tecentriq, Bavencio, Imfinzi, Tevimbra, and Loqtorzi), and common spelling variants encountered in spontaneous reporting. The fixed-dose combination product Opdualag (nivolumab and relatlimab-rmbw) was specifically captured as a distinct entity. Both the DRUGNAME and PROD_AI fields were queried to maximize retrieval sensitivity. To improve the specificity of drug–event attribution, analyses were restricted to reports in which the ICI was designated as the primary suspected (PS) drug. The study population was stratified into four mutually exclusive treatment regimens according to mechanism of action: 1) anti-CTLA-4 monotherapy (ipilimumab); 2) anti-PD-1/L1 monotherapy (nivolumab, pembrolizumab, atezolizumab, avelumab, durvalumab, tislelizumab, and toripalimab); 3) nivolumab plus ipilimumab combination therapy (reports listing both agents as suspect drugs); and 4) nivolumab plus relatlimab combination therapy (reports involving the fixed-dose combination Opdualag or concomitant use of its individual components).

The comparator group comprised all FAERS reports from patients with cancer-related indications who were not treated with any ICI. Specifically, patients were identified through cancer-related MedDRA indication terms recorded in the INDI file, and any report in which an ICI appeared in the prescription was excluded from the comparator cohort. This same comparator group was used consistently across all subgroup analyses to ensure comparability of disproportionality signals across treatment regimens.

### Definition of ocular adverse events

Adverse events were coded using the Preferred Terms (PTs) of the Medical Dictionary for Regulatory Activities (MedDRA, version 28.0) (Sriram and u, 2024; Morley, 2014). In this study, potential oAEs were identified primarily within the System Organ Class (SOC) of “Eye disorders.” To ensure comprehensive retrieval, Standardized MedDRA Queries (SMQs) related to ocular conditions were utilized, including but not limited to Optic nerve disorders, Retinal disorders, Corneal disorders, Scleral disorders, and Ocular motility disorders. Furthermore, although broad search terms for ocular inflammation encompass various conditions such as endophthalmitis, “Uveitis” was evaluated as a distinct outcome in this study to differentiate its specific reporting signal profile from generalized eye inflammation.

### Ophthalmologist-reviewed clinical grouping analysis

To address the clinical fragmentation inherent in PT-level reporting, an additional grouped analysis was performed under ophthalmologist review for the most clinically overlapping inflammatory entities. Related PTs were regrouped into the following custom clinical categories (Sun et al., 2024): Anterior Uveitis Spectrum (including Anterior uveitis, Iritis, and Iridocyclitis) (Iranzo et al., 2022); Posterior Uveitis Spectrum (including Posterior uveitis and Chorioretinitis) (Li et al., 2025); Panuveitis; and (Postow et al., 2018) Overall Uveitis Spectrum (encompassing all uveitis-related PTs). The Optic Neuritis/Neuropathy Spectrum, including Optic neuritis, Optic neuropathy, and Retrobulbar neuritis, was likewise evaluated as a grouped entity. To avoid double counting, case-level deduplication was performed at the PrimaryID level: if a single patient reported multiple PTs within the same clinical category, that case was counted only once for the grouped analysis. The SMQ-level analysis was retained as a pharmacovigilance-oriented framework and was not intended to serve as a clinically comprehensive ophthalmic classification.

### Temporal trend analysis

To evaluate whether the pattern of ICI-associated oAEs has changed over time, temporal trend analyses were conducted. The annual number and proportion of total ICI-associated oAE reports, the annual proportion of uveitis-spectrum reports, the annual distribution by tumor type (melanoma versus non-melanoma), and the annual distribution by ICI regimen were calculated. The Cochran-Armitage trend test was used to assess whether the proportion of uveitis among ICI-associated oAEs showed a statistically significant temporal trend over the study period.

### Statistical analysis

Disproportionality analysis was conducted utilizing a 2 × 2 contingency table to compare the reporting frequency of oAEs between the ICI cohort and the comparator group. To guarantee the robustness of signal detection, a multi-faceted approach was adopted, incorporating both frequentist and Bayesian algorithms: the Reporting Odds Ratio (ROR), the Proportional Reporting Ratio (PRR), and the Information Component (IC). A safety signal was considered statistically significant for ROR when the report count was ≥ 3 and the lower bound of the 95% confidence interval (CI) exceeded 1. Similarly, for PRR, a signal was identified if the report count was ≥ 3, the PRR value was ≥ 2, and the Chi-square (χ^2^) statistic was ≥ 4. Regarding the Bayesian metric, a signal was confirmed if the lower limit of the 95% credibility interval for the IC (IC_025_) was greater than 0. To enhance the specificity of the findings, a “Strong Signal” was defined as any drug-event combination satisfying the detection criteria of at least two of these three algorithms simultaneously. It should be noted that the total number of unique cases (reported in Table 1 and throughout the manuscript) refers to deduplicated patient-level counts based on PrimaryID. Because a single case may report multiple ocular PTs, the sum of individual PT-level event counts exceeds the total number of unique cases. For SMQ-level and clinical category-level analyses, case-level deduplication was applied within each category. All data mining and statistical computations were executed using R software (version 4.3.1) and Microsoft Excel.

**TABLE 1:** Characteristics of ocular adverse event reports associated with immune checkpoint inhibitor regimens.

Characteristic	Anti-PD-1/L1 monotherapy	Nivolumab + ipilimumab combo	Anti-CTLA-4 monotherapy	Nivolumab + relatlimab combo
N	3807	974	445	18
Age, years				
Mean (SD)	65.2 (13.3)	61.5 (12.7)	59.9 (13.2)	77.1 (9.6)
Age groups, n (%)				
Pediatric (<18 years)	13 (0.3%)	1 (0.1%)	0 (0.0%)	0 (0.0%)
Adult (18–65 years)	1196 (31.4%)	465 (47.7%)	196 (44.0%)	2 (11.1%)
Elderly (>65 years)	1537 (40.4%)	331 (34.0%)	119 (26.7%)	13 (72.2%)
Sex, n (%)				
Female	1603 (42.1%)	343 (35.2%)	160 (36.0%)	7 (38.9%)
Male	1869 (49.1%)	562 (57.7%)	227 (51.0%)	10 (55.6%)
Report year, n (%)				
2010–2015	94 (2.5%)	10 (1.0%)	231 (51.9%)	0 (0.0%)
2016–2020	1688 (44.3%)	383 (39.3%)	150 (33.7%)	0 (0.0%)
2021–2025	2015 (52.9%)	580 (59.5%)	38 (8.5%)	18 (100.0%)
Reporter type, n (%)				
Physician	1642 (43.1%)	467 (47.9%)	81 (18.2%)	6 (33.3%)
Pharmacist	236 (6.2%)	46 (4.7%)	14 (3.1%)	2 (11.1%)
Other healthcare	317 (8.3%)	123 (12.6%)	124 (27.9%)	0 (0.0%)
Consumer	1033 (27.1%)	146 (15.0%)	178 (40.0%)	5 (27.8%)
Unknown	73 (1.9%)	0 (0.0%)	21 (4.7%)	0 (0.0%)
Serious outcomes, n (%)				
Death	404 (10.6%)	74 (7.6%)	38 (8.5%)	3 (16.7%)
Hospitalization	1349 (35.4%)	410 (42.1%)	179 (40.2%)	3 (16.7%)
Life-threatening	207 (5.4%)	55 (5.6%)	12 (2.7%)	3 (16.7%)

## Results

### Case characteristics and demographics

This study identified a total of 5,244 unique oAE cases associated with ICIs. Based on the therapeutic regimens, the cohort was categorized into four groups: Anti-PD-1/L1 Monotherapy (n = 3,807), Nivolumab + Ipilimumab Combo (n = 974), Anti-CTLA-4 Monotherapy (n = 445), and the novel Nivolumab + Relatlimab Combo (n = 18). Baseline characteristics are summarized in Table 1. The mean age of patients ranged from 59.9 to 77.1 years across regimens, with the highest mean age observed in the nivolumab plus relatlimab group (77.1 ± 9.6 years). Consistently, this group showed a higher proportion of elderly patients (>65 years, 72.2%), whereas the proportion of elderly patients in the other three groups ranged from 26.7% to 40.4%. Male patients accounted for a larger proportion of reports across all treatment regimens, particularly in the combination therapy groups, representing 57.7% of reports in the nivolumab plus ipilimumab group and 55.6% in the nivolumab plus relatlimab group. With respect to reporting year, most reports for nivolumab plus ipilimumab (59.5%) and all reports for nivolumab plus relatlimab (100%) were submitted between 2021 and 2025, reflecting the more recent clinical adoption of these combination therapies. Among serious outcomes, hospitalization was the most frequently reported event across all regimens, with higher proportions observed in the nivolumab plus ipilimumab group (42.1%) and the anti-CTLA-4 monotherapy group (40.2%). Death and life-threatening outcomes were reported less frequently in all groups.

### Distribution of underlying tumor indications

Among all 5,244 ICI-associated oAE cases, the most frequently reported cancer indication was melanoma (1,748 cases, 33.3%), followed by lung cancer (1,101 cases, 21.0%) and renal cancer (562 cases, 10.7%) (Supplementary Figure S1, panel 1). The indication field was unknown or unspecified in approximately 10%–13% of cases across most subgroups, with a higher missing rate (22.2%) in the small Nivolumab + Relatlimab group. Notably, the distribution of tumor indications varied substantially across oAE categories. Within the uveitis-spectrum subgroup, melanoma accounted for 53.6% of cases (303/565), considerably higher than its overall proportion, whereas lung cancer represented 12.2% and renal cancer 11.0% (Supplementary Figure S1, panel 2). For optic nerve events, melanoma (33.0%) and lung cancer (25.8%) were the leading indications (Supplementary Figure S1, panel 3). In contrast, for corneal events, the relative contribution of melanoma was lower (Supplementary Figure S1, panel 4). Within the Nivolumab + Ipilimumab group, melanoma was predominant at 57.9% (564/974), while the Nivolumab + Relatlimab group showed the highest melanoma proportion at 72.2% (13/18), although based on a very small sample (Supplementary Figure S1, panels 5 and 6). Among the 1,748 melanoma patients with ICI-associated oAEs, 126 (7.18%) received concomitant BRAF/MEK inhibitors, predominantly dabrafenib and trametinib.

### Overall ocular adverse event signals

**FIGURE 1:**
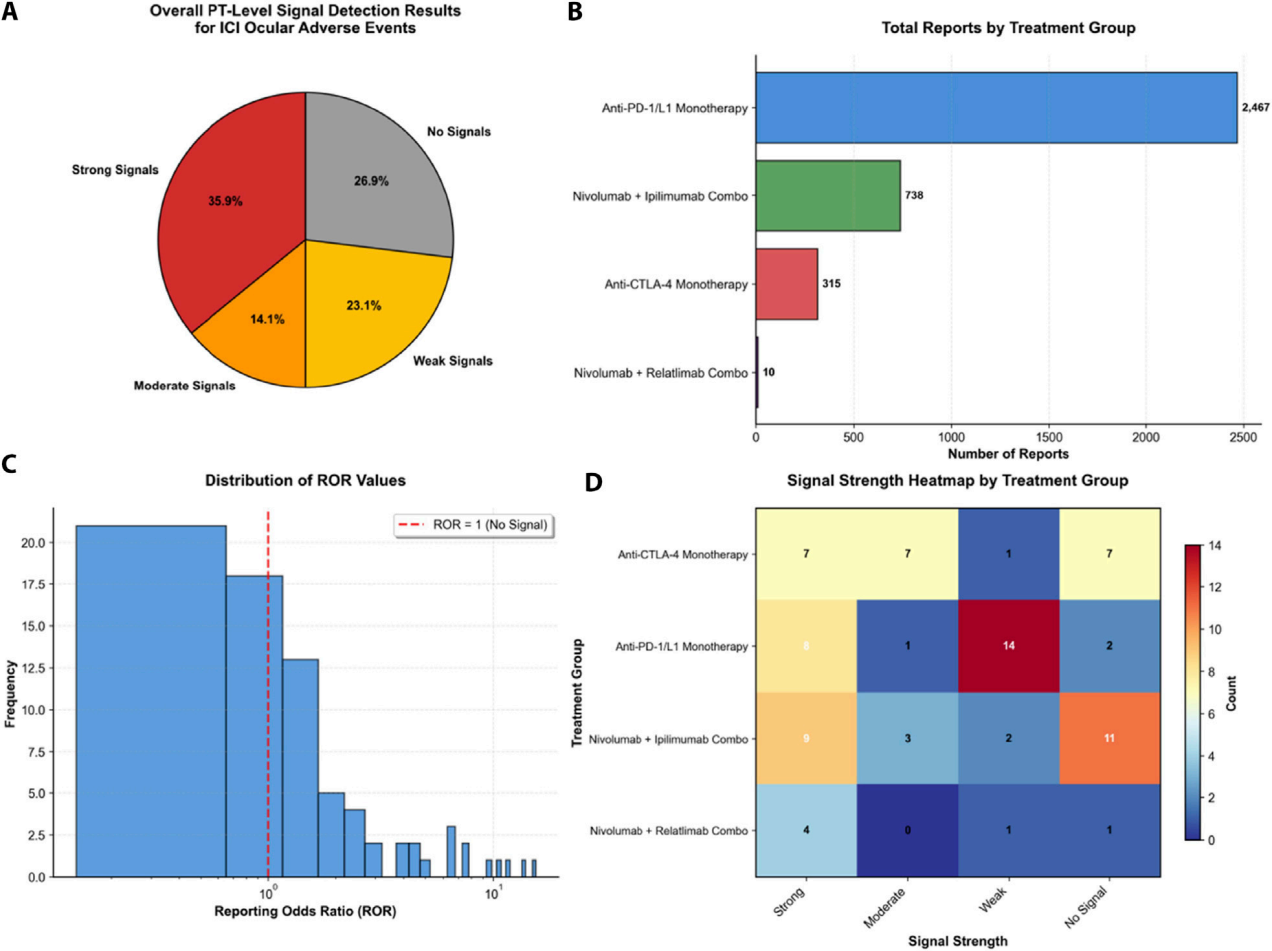
Overall signal detection results for ICI-associated ocular adverse events at the PT level. **(A)** Overall Distribution of oAE Signal Strength; **(B)** Quantitative Distribution of Safety Reports by ICI Regimen; **(C)** Frequency Distribution of Reporting Odds Ratio (ROR) Values; **(D)** Heatmap of Signal Intensity Categorized by Treatment Regimen. The comparator group comprised patients with cancer indications not treated with ICIs.

When aggregating all immune checkpoint inhibitor regimens, multiple statistically significant disproportionality signals were identified for ocular adverse events at the Preferred Term level (Figure 1; Table 2). Overall, 73.1% of PT-level ocular events generated positive disproportionality signals, with Strong Signals accounting for 35.9%, followed by Weak (23.1%) and Moderate (14.1%). By regimen, Anti-PD-1/L1 monotherapy contributed the largest volume of reports (n = 2,467), surpassing Nivolumab + Ipilimumab combination (n = 738) and Anti-CTLA-4 monotherapy (n = 315). The distribution of ROR values demonstrates that the majority of oAEs have ROR > 1, indicating a significant positive disproportionality signal between ICI therapy and ocular adverse events relative to the non-ICI cancer comparator group. The strongest signals, based on reporting odds ratio (ROR) magnitude, were predominantly observed in inflammatory ocular conditions. Uveitis showed the highest signal (ROR 6.04, 95% CI 5.45–6.69), followed by scleritis (ROR 4.85, 95% CI 3.20–7.36) and optic neuritis (ROR 4.59, 95% CI 3.91–5.39). Other inflammatory events, including iridocyclitis (ROR 4.12) and iritis (ROR 3.20), also exhibited robust signals. Among neuro-ophthalmic adverse events, diplopia presented a strong signal (ROR 1.78, 95% CI 1.58–2.00).

**TABLE 2:** Disproportionality analysis signals for the top 10 ocular adverse events (PT level) for all ICIs combined.

PT term	Signal strength	Cases (ICI + AE)	ROR (95% CI)	PRR (95% CI)	IC (95% CI)
Uveitis	Strong	585	6.04 (5.45–6.69)	6.02 (5.43–6.67)	2.04 (1.90–2.18)
Iridocyclitis	Strong	105	4.12 (3.28–5.18)	4.12 (3.28–5.18)	1.68 (1.36–1.99)
Iritis	Strong	39	3.20 (2.23–4.60)	3.20 (2.23–4.59)	1.41 (0.91–1.92)
Optic neuritis	Strong	219	4.59 (3.91–5.39)	4.59 (3.91–5.39)	1.79 (1.57–2.01)
Optic neuropathy	Strong	42	1.46 (1.05–2.01)	1.46 (1.05–2.01)	0.48 (0.02–0.95)
Papilloedema	Strong	88	1.54 (1.23–1.93)	1.54 (1.23–1.93)	0.55 (0.23–0.87)
Retinal detachment	Moderate	140	1.14 (0.96–1.36)	1.14 (0.96–1.36)	0.18 (−0.08–0.43)
Scleritis	Strong	33	4.85 (3.20–7.36)	4.85 (3.20–7.36)	1.84 (1.27–2.41)
Keratitis	Strong	116	1.36 (1.12–1.65)	1.36 (1.12–1.65)	0.40 (0.12–0.67)
Conjunctivitis	Moderate	195	1.03 (0.89–1.19)	1.03 (0.89–1.19)	0.03 (−0.18–0.25)
Diplopia	Strong	329	1.78 (1.58–2.00)	1.78 (1.58–2.00)	0.73 (0.56–0.90)

### Signal profiles by treatment regimen

To characterize the reporting signal profiles of different immunotherapeutic strategies, we analyzed the signal detection results across the four treatment groups at the PT level (Supplementary Figure S2; Table 3). The Anti-CTLA-4 Monotherapy group exhibited prominent signals for uveitic conditions, notably Iridocyclitis, which had an ROR of 11.49 (95% CI 7.20–18.32), and Uveitis with an ROR of 7.63. In comparison, the Anti-PD-1/L1 Monotherapy group, while accounting for the highest volume of reports in the dataset, demonstrated a relatively lower signal strength for Uveitis (ROR 4.37). In the combination therapy cohorts, robust disproportionality signals were observed for inflammatory events. Specifically, the Nivolumab + Ipilimumab Combo group yielded an ROR of 15.44 (95% CI 13.24–18.00) for Uveitis, alongside strong signals for Iridocyclitis (ROR 10.64) and Optic neuritis (ROR 6.63). The Nivolumab + Relatlimab Combo group, though based on a very small number of cases (N = 18), showed notable disproportionality signals for Keratitis (ROR 13.82, 95% CI 3.44–55.61) and Diplopia (ROR 9.59, 95% CI 3.07–29.91); however, given the extremely small sample size, these findings should be interpreted as preliminary, hypothesis-generating signals subject to reporting instability. Among the most robustly associated ocular toxicities, Uveitis, Iridocyclitis, Iritis, Optic neuritis, and Diplopia emerged as the leading PT terms, each identified as a strong signal across 3 distinct treatment categories. The ROR distribution of the top 10 strongest signals, illustrated in Supplementary Figure S3, further highlights the varying signal intensities observed across these regimens.

**TABLE 3:** Signal detection results for ocular adverse events (PT level) by ICI treatment regimen.

Treatment group	PT term	Reports	ROR (95% CI)	PRR (95% CI)	IC (95% CI)
Anti-CTLA-4 monotherapy	Iridocyclitis	19	11.49 (7.20–18.32)	11.47 (7.20–18.28)	3.42 (2.75–4.09)
	Uveitis	48	7.63 (5.70–10.20)	7.60 (5.69–10.15)	2.86 (2.45–3.28)
	Optic neuritis	23	7.43 (4.88–11.29)	7.41 (4.88–11.26)	2.83 (2.23–3.43)
	Iritis	5	6.31 (2.58–15.45)	6.31 (2.58–15.44)	2.61 (1.32–3.90)
	Optic neuropathy	5	2.67 (1.10–6.46)	2.67 (1.10–6.46)	1.40 (0.12–2.67)
	Conjunctivitis	26	2.11 (1.43–3.10)	2.10 (1.43–3.10)	1.06 (0.51–1.62)
	Vision blurred	59	1.36 (1.05–1.76)	1.36 (1.05–1.75)	0.44 (0.07–0.81)
	Chorioretinitis	5	1.95 (0.81–4.72)	1.95 (0.81–4.72)	0.96 (−0.32–2.23)
	Papilloedema	6	1.61 (0.72–3.61)	1.61 (0.72–3.61)	0.69 (−0.47–1.85)
	Diplopia	18	1.50 (0.94–2.38)	1.50 (0.94–2.38)	0.58 (−0.09–1.24)
	Periorbital oedema	4	1.44 (0.54–3.85)	1.44 (0.54–3.84)	0.52 (−0.90–1.94)
	Retinal detachment	10	1.26 (0.67–2.34)	1.26 (0.67–2.34)	0.33 (−0.57–1.23)
	Eye pain	12	1.24 (0.70–2.19)	1.24 (0.70–2.18)	0.31 (−0.51–1.12)
	Photophobia	5	1.01 (0.42–2.43)	1.01 (0.42–2.43)	0.01 (−1.25–1.28)
Anti-PD-1/L1 monotherapy	Scleritis	27	4.96 (3.17–7.76)	4.96 (3.17–7.76)	1.93 (1.32–2.55)
	Uveitis	339	4.37 (3.86–4.95)	4.36 (3.85–4.94)	1.80 (1.63–1.97)
	Optic neuritis	154	4.04 (3.36–4.84)	4.03 (3.36–4.84)	1.72 (1.46–1.97)
	Iritis	27	2.77 (1.82–4.21)	2.77 (1.82–4.20)	1.29 (0.70–1.88)
	Iridocyclitis	50	2.45 (1.81–3.32)	2.45 (1.81–3.32)	1.14 (0.71–1.58)
	Diplopia	250	1.69 (1.48–1.93)	1.69 (1.48–1.93)	0.68 (0.49–0.87)
	Optic neuropathy	34	1.47 (1.03–2.10)	1.47 (1.03–2.10)	0.51 (−0.00–1.02)
	Keratitis	95	1.39 (1.12–1.72)	1.39 (1.12–1.72)	0.43 (0.13–0.74)
	Papilloedema	59	1.29 (0.99–1.69)	1.29 (0.99–1.69)	0.34 (−0.05–0.72)
Nivolumab + ipilimumab combo	Uveitis	198	15.44 (13.24–18.00)	15.32 (13.16–17.85)	3.69 (3.47–3.90)
	Iridocyclitis	36	10.64 (7.50–15.09)	10.62 (7.49–15.06)	3.24 (2.74–3.73)
	Scleritis	6	6.63 (2.88–15.29)	6.63 (2.88–15.29)	2.63 (1.42–3.83)
	Optic neuritis	42	6.63 (4.83–9.09)	6.62 (4.83–9.07)	2.62 (2.17–3.08)
	Iritis	7	4.32 (2.02–9.26)	4.32 (2.02–9.26)	2.05 (0.95–3.15)
	Papilloedema	23	3.03 (1.99–4.59)	3.03 (1.99–4.59)	1.56 (0.96–2.16)
	Diplopia	58	2.36 (1.82–3.06)	2.36 (1.81–3.06)	1.21 (0.83–1.59)
	Retinal detachment	37	2.28 (1.64–3.16)	2.27 (1.64–3.15)	1.16 (0.69–1.63)
	Eye pain	33	1.66 (1.18–2.35)	1.66 (1.18–2.35)	0.72 (0.22–1.22)
	Keratitis	15	1.32 (0.79–2.20)	1.32 (0.79–2.20)	0.40 (−0.34–1.13)
	Conjunctivitis	33	1.31 (0.93–1.84)	1.31 (0.93–1.84)	0.38 (−0.12–0.87)
	Dry eye	41	1.19 (0.87–1.62)	1.19 (0.87–1.62)	0.24 (−0.20–0.69)
Nivolumab + relatlimab combo	Keratitis	2	13.82 (3.44–55.61)	13.74 (3.45–54.79)	3.78 (1.78–5.77)
	Diplopia	3	9.59 (3.07–29.91)	9.50 (3.08–29.33)	3.25 (1.62–4.87)
	Eye pain	1	3.94 (0.55–28.10)	3.93 (0.56–27.85)	1.97 (−0.85–4.80)
	Lacrimation increased	1	2.18 (0.31–15.51)	2.17 (0.31–15.39)	1.12 (−1.70–3.94)

### Signal analysis at the standardized MedDRA query (SMQ) level

**FIGURE 2:**
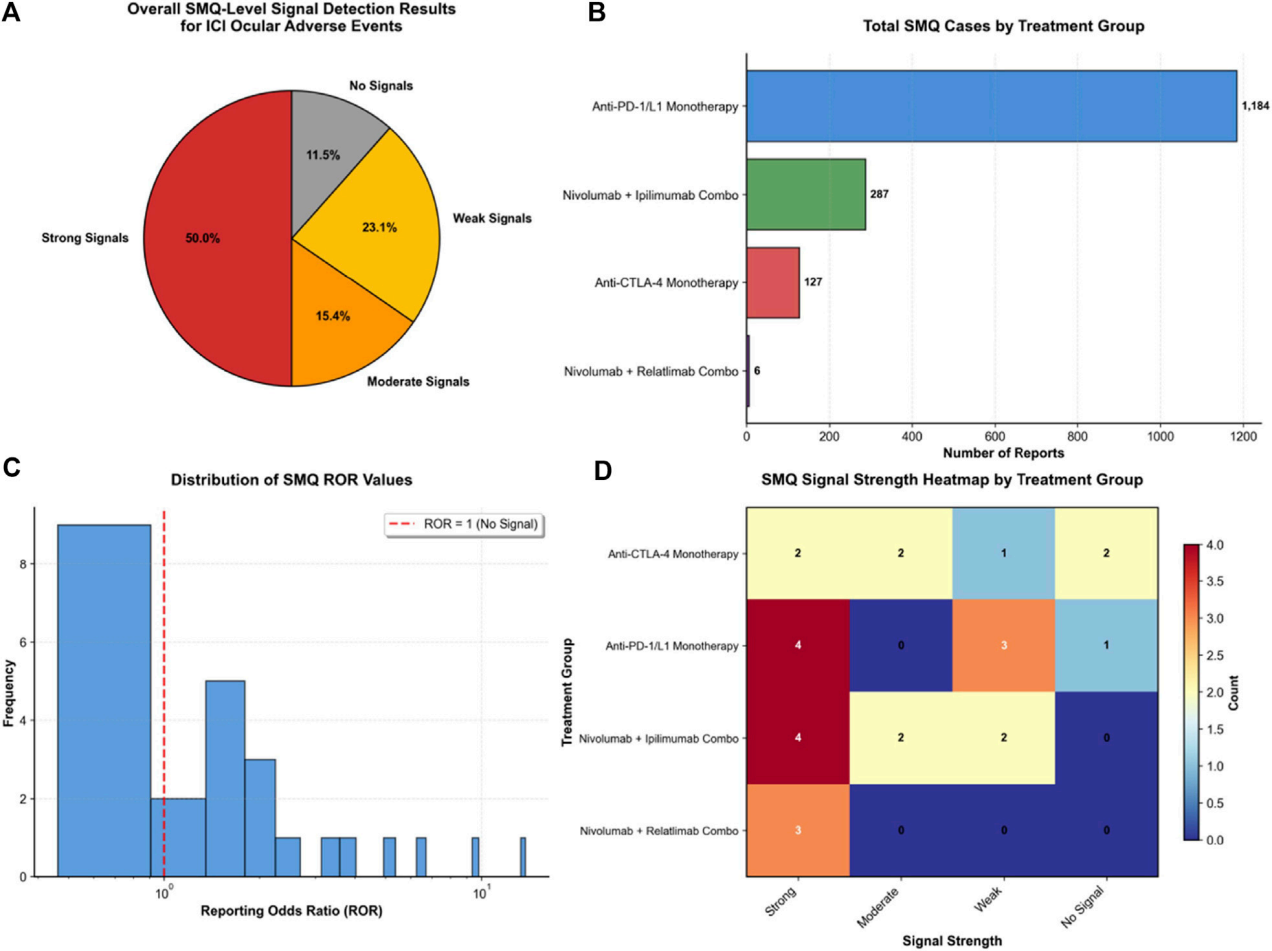
Overall signal detection results for ICI-associated ocular adverse events at the SMQ category level. **(A)** Overall SMQ-Level Signal Detection Results; **(B)** Total SMQ Cases by Treatment Group; **(C)** Distribution of SMQ ROR Values; **(D)** SMQ Signal Strength Heatmap by Treatment Group. SMQ categories were applied as a pharmacovigilance analytical framework and do not represent a clinically comprehensive ophthalmic classification.

We further evaluated these observations at the broader SMQ category level, where 50.0% of all identified SMQ categories were classified as Strong Signals (Figure 2; Supplementary Figure S4). It should be noted that the SMQ classification was applied as a pharmacovigilance-oriented analytical framework and does not represent a clinically comprehensive ophthalmic classification system. The Anti-PD-1/L1 Monotherapy group contributed the largest number of reports (n = 1,184), which were primarily clustered in Optic nerve disorders and Ocular motility disorders (Supplementary Figure S4). A detailed stratification of SMQ categories by regimen revealed distinct reporting profiles (Table 4). For Anti-PD-1/L1 Monotherapy, while Optic nerve disorders were frequent, the strongest disproportionality was observed for Scleral disorders with an ROR of 4.96 (95% CI 3.17–7.76), followed by Optic nerve disorders (ROR 2.11) and Ocular motility disorders (ROR 1.69). The Anti-CTLA-4 Monotherapy group showed a more focused **signal** profile, generating a strong signal primarily for Optic nerve disorders (ROR 3.91) and Conjunctival disorders (ROR 2.11). In the combination therapy cohorts, the Nivolumab + Ipilimumab Combo group exhibited a broader spectrum of strong signals, encompassing Scleral disorders, which reached an ROR of 6.63 (95% CI 2.88–15.29), as well as Optic nerve disorders (ROR 3.53), Ocular motility disorders (ROR 2.36), and Retinal disorders (ROR 1.77). Furthermore, the Nivolumab + Relatlimab Combo showed elevated disproportionality at this aggregate level; notably, Corneal disorders yielded an ROR of 13.82, followed by Ocular motility disorders (ROR 9.59) and Lacrimal disorders (ROR 2.18), all of which were classified as Strong Signals, although these estimates carry wide confidence intervals due to the very limited case count. Consistent with these findings, the boxplot analysis in Supplementary Figure S4 provided a descriptive overview of the SMQ ROR distributions, showing a higher median ROR for the combination therapies compared to the monotherapy groups in this dataset.

**TABLE 4:** Signal detection results for ocular adverse event SMQ categories by ICI treatment regimen.

Treatment group	SMQ category	Reports	ROR (95% CI)	PRR (95% CI)	IC (95% CI)	Signal strength
Anti-CTLA-4 monotherapy	Periorbital and eyelid disorders	13	0.78 (0.45–1.34)	0.78 (0.45–1.34)	−0.36 (−1.14–0.43)	No signal
	Corneal disorders	4	0.72 (0.27–1.92)	0.72 (0.27–1.92)	−0.47 (−1.89–0.95)	No signal
	Optic nerve disorders	34	3.91 (2.78–5.50)	3.91 (2.78–5.50)	1.94 (1.45–2.43)	Strong
	Conjunctival disorders	26	2.11 (1.43–3.10)	2.10 (1.43–3.10)	1.06 (0.51–1.62)	Strong
	Lacrimal disorders	17	0.49 (0.31–0.79)	0.49 (0.31–0.80)	−1.01 (−1.70–0.33)	Weak
	Ocular motility disorders	18	1.50 (0.94–2.38)	1.50 (0.94–2.38)	0.58 (−0.09–1.24)	Moderate
	Retinal disorders	15	1.43 (0.86–2.37)	1.43 (0.86–2.37)	0.51 (−0.22–1.24)	Moderate
Anti-PD-1/L1 monotherapy	Conjunctival disorders	136	0.89 (0.75–1.06)	0.89 (0.75–1.06)	−0.15 (−0.40–0.10)	No signal
	Scleral disorders	27	4.96 (3.17–7.76)	4.96 (3.17–7.76)	1.93 (1.32–2.55)	Strong
	Optic nerve disorders	252	2.11 (1.85–2.42)	2.11 (1.85–2.42)	0.96 (0.77–1.15)	Strong
	Ocular motility disorders	250	1.69 (1.48–1.93)	1.69 (1.48–1.93)	0.68 (0.49–0.87)	Strong
	Corneal disorders	95	1.39 (1.12–1.72)	1.39 (1.12–1.72)	0.43 (0.13–0.74)	Strong
	Retinal disorders	116	0.81 (0.67–0.98)	0.81 (0.67–0.98)	−0.28 (−0.55–0.01)	Weak
	Lacrimal disorders	213	0.50 (0.44–0.58)	0.50 (0.44–0.58)	−0.94 (−1.13–0.74)	Weak
	Periorbital and eyelid disorders	95	0.46 (0.38–0.57)	0.46 (0.38–0.57)	−1.05 (−1.35–0.76)	Weak
Nivolumab + ipilimumab combo	Scleral disorders	6	6.63 (2.88–15.29)	6.63 (2.88–15.29)	2.63 (1.42–3.83)	Strong
	Optic nerve disorders	70	3.53 (2.78–4.49)	3.53 (2.78–4.49)	1.77 (1.43–2.12)	Strong
	Ocular motility disorders	58	2.36 (1.82–3.06)	2.36 (1.81–3.06)	1.21 (0.83–1.59)	Strong
	Retinal disorders	42	1.77 (1.30–2.40)	1.77 (1.30–2.40)	0.81 (0.36–1.25)	Strong
	Lacrimal disorders	46	0.65 (0.49–0.87)	0.65 (0.49–0.87)	−0.61 (−1.03–0.19)	Weak
	Periorbital and eyelid disorders	17	0.50 (0.31–0.80)	0.50 (0.31–0.80)	−1.00 (−1.68–0.31)	Weak
	Corneal disorders	15	1.32 (0.79–2.20)	1.32 (0.79–2.20)	0.40 (−0.34–1.13)	Moderate
	Conjunctival disorders	33	1.31 (0.93–1.84)	1.31 (0.93–1.84)	0.38 (−0.12–0.87)	Moderate
Nivolumab + relatlimab combo	Corneal disorders	2	13.82 (3.44–55.61)	13.74 (3.45–54.79)	3.78 (1.78–5.77)	Strong
	Ocular motility disorders	3	9.59 (3.07–29.91)	9.50 (3.08–29.33)	3.25 (1.62–4.87)	Strong
	Lacrimal disorders	1	2.18 (0.31–15.51)	2.17 (0.31–15.39)	1.12 (−1.70–3.94)	Strong

### Ophthalmologist-reviewed clinical grouping analysis of uveitis and optic neuritis/neuropathy spectra

To provide a clinically meaningful evaluation of overlapping uveitis-related PTs, a grouped analysis was performed with case-level deduplication under ophthalmologist review (Supplementary Tables S1,2). For all ICIs combined, the Anterior Uveitis Spectrum (comprising Iritis and Iridocyclitis; the distinct MedDRA PT “Anterior uveitis” yielded no cases in this dataset) showed a strong positive signal (n = 138; ROR 4.93, 95% CI 4.02–6.05; IC025 = 1.60). The Posterior Uveitis Spectrum (comprising Chorioretinitis; the distinct MedDRA PT “Posterior uveitis” yielded no cases) also showed a positive signal (n = 27; ROR 5.32, 95% CI 3.33–8.50; IC025 = 1.34). No cases of Panuveitis were identified. The Overall Uveitis Spectrum, aggregating all uveitis-related PTs, yielded a robust positive signal (n = 565; ROR 5.23, 95% CI 4.72–5.79; IC025 = 1.78).

When stratified by treatment regimen (Supplementary Table S2), the Anterior Uveitis Spectrum signal was strongest in the Nivolumab + Ipilimumab group (n = 43; ROR 11.56), followed by Anti-CTLA-4 Monotherapy (n = 18; ROR 9.89), whereas Anti-PD-1/L1 Monotherapy showed a lower but still positive signal (n = 77; ROR 3.44). For the Posterior Uveitis Spectrum, positive signals were observed for Anti-CTLA-4 Monotherapy (n = 5; ROR 15.16), Anti-PD-1/L1 Monotherapy (n = 18; ROR 4.43), and Nivolumab + Ipilimumab (n = 4; ROR 5.93), although these estimates were based on small case numbers. For the Overall Uveitis Spectrum, the Nivolumab + Ipilimumab group demonstrated the strongest signal (n = 179; ROR 12.51), followed by Anti-CTLA-4 Monotherapy (n = 58; ROR 8.27) and Anti-PD-1/L1 Monotherapy (n = 328; ROR 3.79). No uveitis-spectrum cases were reported in the Nivolumab + Relatlimab group.

In addition, the Optic Neuritis/Neuropathy Spectrum, comprising Optic neuritis, Optic neuropathy, and Retrobulbar neuritis, was evaluated as a grouped entity with case-level deduplication. Across all ICI regimens combined, this spectrum showed a positive disproportionality signal compared with non-ICI cancer controls (n = 236; ROR 5.03, 95% CI 4.30–5.88; PRR 5.02, 95% CI 4.29–5.87; IC = 1.87, IC025 = 1.68). In regimen-stratified analyses, positive signals were observed for anti-CTLA-4 monotherapy (n = 21; ROR 6.88, 95% CI 4.44–10.66; IC025 = 2.11), anti-PD-1/L1 monotherapy (n = 177; ROR 4.71, 95% CI 3.96–5.60; IC025 = 1.65), and nivolumab plus ipilimumab combination therapy (n = 38; ROR 6.09, 95% CI 4.37–8.47; IC025 = 2.05). No optic neuritis/neuropathy-spectrum cases were reported in the nivolumab plus relatlimab group (Supplementary Tables S3, 4).

### Temporal trend analysis

Temporal analyses revealed a steady increase in the absolute number of ICI-associated oAE reports over the study period, rising from fewer than 100 annual reports before 2015 to over 650 by 2024 (Supplementary Figure S5, panel A). The proportion of uveitis-spectrum events among all ICI-associated oAEs peaked in the early years of reporting (approximately 14%–15% in 2014–2015), then declined to approximately 8%–9% in 2022–2024. The Cochran-Armitage trend test showed a non-significant downward trend (Z = −1.399, p = 0.162) (Supplementary Figure S5, panel A).

The annual distribution of reports by ICI regimen shifted substantially over time (Supplementary Figure S5, panels B and C). Anti-CTLA-4 monotherapy dominated oAE reporting in 2012–2013 (accounting for nearly 100% of reports) but declined rapidly after 2014 as anti-PD-1/L1 agents became the predominant treatment. Nivolumab + Ipilimumab combination reports emerged from 2015 and contributed a stable proportion from 2017 onward. Nivolumab + Relatlimab reports appeared only from 2022, consistent with its recent approval.

With respect to tumor indication, melanoma accounted for over 95% of ICI-associated oAE cases in the earliest years (2012–2013) but declined progressively to approximately 25% by 2023–2024, paralleling the expansion of ICI approvals to lung cancer, renal cancer, and other non-melanoma indications (Supplementary Figure S5, panel D). This temporal decline in the melanoma proportion coincided with the observed decrease in the uveitis proportion, providing indirect support for the hypothesis that the underlying tumor biology, particularly shared melanocytic antigens in melanoma, is a contributing determinant of the uveitis signal.

## Discussion

This pharmacovigilance study, utilizing FAERS data up to 2025 Q2, confirms that ICIs are associated with a significant and diverse spectrum of inflammatory oAEs, and reveals that the disproportionality signals are substantially magnified under combination therapy. Our principal findings reaffirm that uveitis, optic neuritis/neuropathy-spectrum events, and scleritis are the most consistently reported oAEs, aligning with established literature.

Our analysis, leveraging the most current data, extends the foundational work of Fang et al. and Bomze et al. (Fang et al., 2019; Bomze et al., 2022). While those studies first identified these signals using data from 2018–2019, our analysis demonstrates that these signals remain strong and persistent. More importantly, our study provides the first large-scale, real-world signal analysis for the novel anti-LAG-3 combination, Nivolumab + Relatlimab. Although based on a very small number of cases (N = 18), we observed notable disproportionality signals for Keratitis (ROR 13.82) and Diplopia (ROR 9.59) that met criteria for ROR, PRR, and IC. These findings should be regarded as preliminary, hypothesis-generating observations rather than evidence of a confirmed distinct toxicity profile, and require validation in larger datasets as clinical experience with this regimen expands.

It is well established that combination ICI therapy is associated with higher rates of immune-related adverse events compared with monotherapy across multiple organ systems (Da et al., 2020; Park et al., 2020). Specifically for ocular events, a systematic review and meta-analysis demonstrated that PD-1 plus CTLA-4 combination significantly increased the odds of ophthalmic immune-related adverse events compared with monotherapy (OR 4.52, p < 0.01) (Hou et al., 2022). Our findings are consistent with this known pattern and provide an updated quantification using the most recent FAERS data. For example, the ROR for uveitis with the Nivolumab plus Ipilimumab (anti-PD-1 + anti-CTLA-4) regimen reached 15.44, far exceeding the 4.37 ROR observed with anti-PD-1/L1 monotherapy. This synergistic toxicity is not merely an additive effect but is rooted in well-characterized immunological mechanisms (Da et al., 2020; Park et al., 2020). CTLA-4 and PD-1 are two critical but non-redundant immune brakes. Anti-CTLA-4 (such as Ipilimumab) primarily acts during the priming phase in lymph nodes, blocking the initial inhibition of T-cell activation and proliferation, thereby greatly expanding the T-cell repertoire, particularly CD4^+^ T cells (Rosskopf et al., 2019). In contrast, anti-PD-1 (such as Nivolumab) primarily acts during the effector phase within the tumor microenvironment and peripheral tissues, including the eye, reactivating exhausted T cells (especially CD8^+^ T cells) that have already infiltrated the tissue (Jiang et al., 2021; Veluswamy and Bruder, 2018).

Therefore, the dual blockade of CTLA-4 and PD-1 is a ‘double-hit’. It ‘manufactures’ more activated T cells in the lymph nodes while simultaneously ‘releasing’ their killing function in the periphery, leading to a broader, more durable, and more intense systemic T-cell-mediated immune activation (Zhang et al., 2021; Wei et al., 2017). This is the common cause for both superior anti-tumor efficacy and more severe irAEs, including oAEs. Specific to the eye, this T-cell-mediated inflammation is not random. A widely accepted mechanism is ‘antigen sharing.’ (Chen S. et al., 2024; Blum et al., 2023) For instance, the high proportion of uveitis reports in melanoma patients is thought to occur because T cells, activated to attack melanoma-associated antigens (like gp100, tyrosinase) on tumor cells, also cross-react with similar antigens expressed on uveal melanocytes in the eye (Przybyla et al., 2021). ICI therapy breaks the eye’s immune privilege, allowing this autoimmune attack to occur. Furthermore, this T-cell activation releases a flood of pro-inflammatory cytokines, such as interferon-gamma (IFN-γ), tumor necrosis factor-alpha (TNF-α), and interleukins IL-6 and IL-17 (Yameny, 2025). This specific cytokine profile is highly consistent with the pathophysiology of non-infectious uveitis, confirming that ICI-induced oAEs are targeted autoimmune processes driven by specific T-cell subsets (like Th1 and Th17), rather than a broad cytokine storm (Ayass et al., 2023).

Our tumor indication analysis provides further support for the antigen-sharing hypothesis. Melanoma accounted for 53.6% of uveitis-spectrum cases, substantially exceeding its 33.3% overall proportion among all ICI-associated oAE reports, whereas its representation in corneal events was comparatively lower. The temporal trend analysis showed that as ICI use expanded beyond melanoma to lung cancer, renal cancer, and other indications, the melanoma proportion among oAE cases declined from over 95% (2012–2013) to approximately 25% (2023–2024), and the uveitis proportion showed a concurrent downward trend (Cochran-Armitage Z = −1.399, p = 0.162). Although not statistically significant, this parallel decline is consistent with the hypothesis that underlying tumor biology, particularly shared melanocytic antigens in melanoma, is a contributing determinant of the uveitis signal. However, an important confounding factor should be noted: among the 1,748 melanoma patients with ICI-associated oAEs, 126 (7.18%) received concomitant BRAF/MEK inhibitors (predominantly dabrafenib and trametinib), which themselves carry well-documented ocular toxicity including uveitis and retinopathy (Mettler et al., 2021; Dimitriou et al., 2021). This co-medication may have partially contributed to the elevated uveitis signal observed in the melanoma subgroup.

Regarding the keratitis signal observed with the Nivolumab + Relatlimab combination, several important caveats warrant discussion. The MedDRA PT “Keratitis” as recorded in FAERS encompasses a heterogeneous group of conditions, including immune-mediated keratitis, dry-eye-related epithelial disease, neurotrophic keratopathy, infectious keratitis, and exposure-related corneal disease, which the database cannot reliably distinguish. Additionally, patients receiving this regimen may differ systematically from other ICI cohorts in age (mean 77.1 years), tumor type (72.2% melanoma), prior treatment exposure, and comorbidity profile, all of which represent potential unmeasured confounders. The extremely small sample size (N = 18) further increases the likelihood of reporting instability and chance findings. Whether blocking LAG-3 in combination with PD-1 activates distinct T-cell subsets that preferentially target corneal antigens remains entirely speculative and requires dedicated immunological investigation (Wu et al., 2024).

The findings of this study have implications for clinical practice, though they should be interpreted within the constraints of spontaneous reporting data. The high ROR values, such as 15.44 for uveitis with Nivolumab plus Ipilimumab, represent strong disproportionality signals that prioritize these events for pharmacovigilance attention. Ocular symptoms in patients receiving ICI therapy, including new-onset blurred vision, eye pain, floaters, or diplopia, warrant prompt ophthalmologic assessment. High-risk patients, including those with melanoma, prior ocular inflammation, autoimmune disease, or receiving combination ICI therapy, may benefit from closer surveillance, particularly during the first 6 months of treatment (Bomze et al., 2022). Management decisions regarding continuation, interruption, or discontinuation of ICI therapy in the setting of oAEs should be individualized and determined through multidisciplinary discussion among the ophthalmologist (preferably a uveitis specialist), oncologist, and patient, carefully balancing the risk of vision-threatening inflammation against cancer control (Shahzad et al., 2021).

This study has several limitations inherent to FAERS-based analyses that should be considered when interpreting the results. First, FAERS is subject to under-reporting, selective reporting, and reporting bias (including the Weber effect for newly approved agents), and lacks a reliable denominator; therefore, true incidence rates cannot be estimated and causal inferences cannot be established. Disproportionality metrics (ROR, PRR, IC) identify reporting signals, not absolute or relative risk. Second, key clinical information, including drug dose, treatment duration, tumor type, comorbidities, concomitant medications, and time-to-onset, is frequently incomplete, limiting adjustment for confounding factors. Although the indication field was available and analyzable, tumor indication data were missing or unspecified in approximately 10%–13% of cases (and 22.2% in the Nivolumab + Relatlimab group), and the cancer type distribution was not matched between the ICI and non-ICI comparator cohorts. Consequently, it remains difficult to fully disentangle whether the observed signals are driven primarily by treatment regimen, by tumor biology, or by both. Third, although analyses were restricted to reports in which ICIs were designated as the primary suspected drugs, residual confounding by indication cannot be fully excluded. Fourth, several closely related PTs (such as uveitis, iritis, and iridocyclitis, as well as optic neuritis and optic neuropathy) appeared as separate entries in our PT-level analyses. This reflects MedDRA coding practices and spontaneous reporting conventions rather than a conceptual overlap introduced by the investigators (Brown et al., 1999). To address this, we performed additional ophthalmologist-reviewed grouped analyses; however, the SMQ-level classification retained as a pharmacovigilance framework does not fully capture the clinical spectrum of ocular irAEs. Fifth, the number of reports for the Nivolumab + Relatlimab combination was very small (N = 18); the disproportionality signals observed for this regimen should be regarded as early signals requiring confirmation. A dose-response analysis was not feasible because the FAERS dose field is frequently missing or recorded as free text, and most current ICI regimens employ approved fixed-dose schedules rather than individualized dose titration. Accordingly, the results of this study should be viewed as hypothesis-generating rather than definitive.

## Conclusion

In this large-scale, updated pharmacovigilance analysis, we confirm that ICI therapy is associated with significant disproportionality signals for serious, inflammatory ocular adverse events, primarily uveitis, optic neuritis, and scleritis. Consistent with prior literature, we demonstrate a substantial magnification of these signals with Nivolumab + Ipilimumab combination therapy and provide updated quantitative estimates from the most recent FAERS data. We additionally report preliminary signals for keratitis and diplopia associated with the Nivolumab + Relatlimab combination, though these require confirmation given the very small sample size. Tumor indication analysis revealed that melanoma was disproportionately represented among uveitis-spectrum cases, supporting the role of shared melanocytic antigens in driving this specific signal. These findings highlight a clear, regimen-dependent reporting pattern and underscore the necessity for prompt ophthalmologic assessment of ocular symptoms in patients undergoing ICI treatment, with individualized, multidisciplinary management for those on combination regimens.

## Data Availability

The original contributions presented in the study are included in the article/Supplementary Material, further inquiries can be directed to the corresponding author.
