# Supplementary material for: Ocular adverse events associated with immune checkpoint inhibitors: a pharmacovigilance analysis of the FAERS database
Source: Front Pharmacol. 2026 Jul 27;17:1808874. doi: 10.3389/fphar.2026.1808874 (PMC13454064; doi:10.3389/fphar.2026.1808874)
Supplement: Supplementary file 1 [file Supplementaryfile1.docx]

**Supplementary Table S1. Disproportionality signal analysis of uveitis anatomical subtypes in ICI-treated patients versus non-ICI cancer controls (FAERS).**

| **Uveitis Subtype** | **PT Terms Included** | **n (ICI cases)** | **ROR (95% CI)** | **PRR (95% CI)** | **IC (IC025)** | **Fisher's p** | **Signal** |
| --- | --- | --- | --- | --- | --- | --- | --- |
| **Anterior Uveitis Spectrum** | Anterior uveitis, Iritis, Iridocyclitis | 138 | 4.93 (4.02–6.05) | 4.93 (4.02–6.04) | 1.85 (1.60) | 0.0000 | **Positive** |
| Posterior Uveitis Spectrum | Posterior uveitis, Chorioretinitis | 27 | 5.32 (3.33–8.50) | 5.32 (3.33–8.50) | 1.93 (1.34) | 0.0000 | **Positive** |
| Panuveitis | Panuveitis | 0 | — | — | — | — | None |
| **Overall Uveitis Spectrum** | Uveitis, Anterior uveitis, Iritis, Iridocyclitis, Posterior uveitis, Chorioretinitis, Panuveitis | 565 | 5.23 (4.72–5.79) | 5.22 (4.71–5.78) | 1.91 (1.78) | 0.0000 | **Positive** |

Signals were assessed using Reporting Odds Ratio (ROR), Proportional Reporting Ratio (PRR), and Information Component (IC). Case-level deduplication was applied at the Primary ID level within each subtype. A positive signal was defined as meeting at least two of three criteria: ROR 95% CI lower bound > 1, PRR 95% CI lower bound > 1, and IC025 > 0, with n ≥ 3.

*Abbreviations: ICI, immune checkpoint inhibitor; ROR, Reporting Odds Ratio; PRR, Proportional Reporting Ratio; IC, Information Component; IC025, lower boundary of the 95% confidence interval for IC; n, number of unique cases (deduplicated at PrimaryID level). Note: The distinct MedDRA PTs "Anterior uveitis," "Posterior uveitis," and "Panuveitis" were not identified in the current dataset and are counted as 0; the Anterior Uveitis Spectrum therefore comprises Iritis and Iridocyclitis, and the Posterior Uveitis Spectrum comprises Chorioretinitis only.*

**Supplementary Table S2. Uveitis subtype disproportionality signals stratified by ICI treatment regimen (FAERS).**

| **Uveitis Subtype** | **All ICI** | | | **Anti-CTLA-4 Monotherapy** | | | **Anti-PD-1/L1 Monotherapy** | | | **Nivolumab+ Ipilimumab** | | | **Nivolumab+ Relatlimab** | | |
| --- | --- | --- | --- | --- | --- | --- | --- | --- | --- | --- | --- | --- | --- | --- | --- |
|  | **n** | **ROR (95% CI)** | **IC025** | **n** | **ROR (95% CI)** | **IC025** | **n** | **ROR (95% CI)** | **IC025** | **n** | **ROR (95% CI)** | **IC025** | **n** | **ROR (95% CI)** | **IC025** |
| **Anterior Uveitis** | **138** | **4.93 (4.02–6.05)** | **1.60** | **18** | **9.89 (6.14–15.94)** | **2.56** | **77** | **3.44 (2.67–4.42)** | **1.20** | **43** | **11.56 (8.38–15.94)** | **2.90** | 0 | — | — |
| **Posterior Uveitis** | **27** | **5.32 (3.33–8.50)** | **1.34** | **5** | **15.16 (6.04–38.01)** | **2.56** | **18** | **4.43 (2.59–7.60)** | **1.12** | **4** | **5.93 (2.14–16.41)** | **1.12** | 0 | — | — |
| **Panuveitis** | 0 | — | — | 0 | — | — | 0 | — | — | 0 | — | — | 0 | — | — |
| **Overall Uveitis Spectrum** | **565** | **5.23 (4.72–5.79)** | **1.78** | **58** | **8.27 (6.35–10.78)** | **2.60** | **328** | **3.79 (3.35–4.29)** | **1.48** | **179** | **12.51 (10.67–14.66)** | **3.21** | 0 | — | — |

Each treatment group was compared against the same non-ICI cancer patient comparator population. Case-level deduplication was applied within each subtype. Bold values indicate positive disproportionality signals.

*Abbreviations: ICI, immune checkpoint inhibitor; ROR, Reporting Odds Ratio (95% CI); IC025, lower 95% confidence boundary of the Information Component; n, number of unique cases (deduplicated at PrimaryID level). "—" denotes insufficient data (n = 0 or undefined signal metrics). The Nivolumab + Relatlimab group showed 0 uveitis-spectrum cases across all subtypes.*

**Supplementary Table S3. Disproportionality signal analysis of optic neuritis/neuropathy spectrum in ICI-treated patients vs. non-ICI cancer controls (FAERS)**

| **Ophthalmic Spectrum** | **PT Terms Included** | **n (ICI cases)** | **ROR (95% CI)** | **PRR (95% CI)** | **IC (IC025)** | **Fisher's p** | **Signal** |
| --- | --- | --- | --- | --- | --- | --- | --- |
| **Optic Neuritis/Neuropathy Spectrum** | Optic neuritis, Optic neuropathy, Retrobulbar neuritis | 236 | 5.03 (4.30–5.88) | 5.02 (4.29–5.87) | 1.87 (1.68) | 0.0000 | **Positive** |

*Abbreviations: ICI, immune checkpoint inhibitor; ROR, Reporting Odds Ratio; PRR, Proportional Reporting Ratio; IC, Information Component; IC025, lower boundary of the 95% confidence interval for IC; n, number of unique cases (deduplicated at PrimaryID level). A positive signal is defined as meeting at least two of three criteria: ROR 95%CI lower bound >1, PRR 95%CI lower bound >1, and IC025 >0, with n >= 3. * Reporting instability: n < 5 with IC025 marginally above 0 (0 < IC025 < 0.5). Note: Optic atrophy was not included in the spectrum to focus purely on acute inflammatory/injury events.*

**Supplementary Table S4. Optic neuritis/neuropathy spectrum signals stratified by ICI treatment regimen (FAERS)**

| **Ophthalmic Spectrum** | **All ICI** | | | **Anti-CTLA-4 Monotherapy** | | | **Anti-PD-1/L1 Monotherapy** | | | **Nivolumab+ Ipilimumab** | | | **Nivolumab+ Relatlimab** | | |
| --- | --- | --- | --- | --- | --- | --- | --- | --- | --- | --- | --- | --- | --- | --- | --- |
|  | **n** | **ROR (95% CI)** | **IC025** | **n** | **ROR (95% CI)** | **IC025** | **n** | **ROR (95% CI)** | **IC025** | **n** | **ROR (95% CI)** | **IC025** | **n** | **ROR (95% CI)** | **IC025** |
| **Optic Neuritis/Neuropathy Spectrum** | **236** | **5.03 (4.30–5.88)** | **1.68** | **21** | **6.88 (4.44–10.66)** | **2.11** | **177** | **4.71 (3.96–5.60)** | **1.65** | **38** | **6.09 (4.37–8.47)** | **2.05** | 0 | — | — |

*Abbreviations: ICI, immune checkpoint inhibitor; ROR, Reporting Odds Ratio (95% CI); IC025, lower 95% confidence boundary of the Information Component. Bold cells with green shading indicate positive disproportionality signals. * Reporting instability flag: n < 5, IC025 marginally above 0. '—' denotes insufficient data (n = 0 or undefined signal metrics).*
